# Synergistic effects of titanium dioxide nanoparticles and microplastics on lentil seeds by a non-invasive biospeckle optical coherence tomography

**DOI:** 10.3389/fpls.2026.1718010

**Published:** 2026-02-23

**Authors:** Lavista Tyagi, Hirofumi Kadono, Uma Maheswari Rajagopalan

**Affiliations:** 1 Graduate School of Science and Engineering, Saitama University, Saitama, Japan; 2 Innovative Global Program, Shibaura Institute of Technology, Tokyo, Japan

**Keywords:** dynamic optical coherence tomography, nanotoxicity, plant stress response, seed germination, speckle

## Abstract

Titanium dioxide nanoparticles (TiO_2_ NPs) are used in agriculture, cosmetics, energy, and environmental applications, necessitating advanced methods to evaluate their effects on biological systems such as plant growth. This study demonstrates the use of biospeckle optical coherence tomography (bOCT), a novel and non-invasive technique, to rapidly assess the size and concentration-dependent impacts of TiO_2_ NPs as well as the synergistic effects of TiO_2_ NPs and polyethylene microplastics (PEMPs) on lentil (*Lens culinaris)* seeds. The primary objective was to validate bOCT as a rapid and non-invasive tool for assessing NPs-induced biological responses in plants. Seeds were treated with TiO_2_ particles (<5 µm,<100 nm, 21 nm) at concentrations of 0, 25, 100, and 200 mg/L. The sizes were selected based on commercially available TiO_2_ NPs grades in industrial and agricultural applications. For synergy experiments, TiO_2_ NPs (21 nm) at 25 and 100 mg/L were combined with PEMPs (744–4990 nm) at concentration-based ratios of 1:1 and 1:2. A swept-source OCT system (central wavelength: 1.3 µm; bandwidth: 125 nm; sweep frequency: 20 kHz), acquired OCT structural images at 12.5 frames per second and biospeckle images were calculated as the ratio of the standard deviation to the mean of 100 OCT structural images over an 8-second interval, at 0, 5, 10, and 20 hours (h) post-exposure. Smaller TiO_2_ NPs (<100 nm) enhanced internal activity at lower concentrations (25 mg/L), while larger particles (<5 µm) exhibited similar effects at higher concentrations (200 mg/L). These observations were qualitatively consistent with conventional physiological measurements, including germination rates, growth parameters, and antioxidative enzyme activities recorded over 7 days. The co-application of TiO_2_ NPs and PEMPs at a 1:1 ratio alleviated the reduction in internal activity caused by PEMPs alone, while the 1:2 ratio led to a significant decrease in biospeckle contrast, indicating suppressed internal seed activity. bOCT successfully detected early biological responses of TiO_2_ NPs within 20 h, demonstrating its efficiency compared to the conventional methods. The ability of bOCT to monitor dynamic internal changes highlights its potential as a rapid tool for assessing NPs as well as their synergistic effects with polyethylene microplastics on plants.

## 1 Introduction

Nanomaterials ranging from 1 to 100 nanometers in size have attracted significant scientific interest because their properties often differ from those of their bulk counterparts. These differences arise from unique quantum size effects and a high surface area-to-volume ratio. Nanomaterials can be broadly classified into carbon-based, metal-based, and metal oxide nanoparticles, as well as non-metallic substances like silica and polymers (e.g., polystyrene, PLGA). Each class exhibits distinct physical and chemical behaviors suitable for electronics, medicine, and environmental science (Sajid, 2022; Mekuye and Abera, 2023).

Among these nanomaterials, metal oxide nanoparticles (NPs) have gained prominence due to their stability and surface reactivity, which enable both durability and catalytic activity in various applications. Titanium dioxide (TiO_2_) NPs are particularly important as they are widely employed in industrial and consumer products, including paints, sunscreens, photocatalysis, and wastewater treatment (Weir et al., 2012). TiO_2_ NPs have desirable characteristics such as a high refractive index, chemical inertness, and relatively low toxicity for biomedical applications. However, these very properties also imply that when they enter the environment, they may not degrade easily, leading to accumulation over time (Bevacqua et al., 2023).

The extensive utilization and subsequent disposal of products containing TiO_2_ NPs result in their gradual release into the environment. These NPs can enter various environmental media such as soil, water, and air through mechanisms like industrial effluent, runoff, or atmospheric deposition. In the environment, they may ultimately be taken up by plants, which are key components of terrestrial ecosystems and serve as primary producers within the food chain. The accumulation of TiO_2_ NPs in soil raises questions regarding their impacts on plant physiology, particularly in terms of nutrient absorption and overall growth (Zahra et al., 2020).

Recent studies have also focused on the impact of TiO_2_ NPs on seed germination and plant health. Li et al. observed that *Amaranthus mangostanus* seeds exposed to higher concentrations of TiO_2_ NPs after one week exhibited a significant decrease in both germination rate and root growth, accompanied by increased reactive oxygen species (ROS) generation (Li CC. et al., 2022). However, Basahi M. reported a significant improvement after 50 mg/L exposure to TiO_2_ NPs in pea seed germination after observing 1 to 5 days (Basahi, 2021). Additionally, various researchers have reported TiO_2_ as a salt, heat, and heavy metal stress mitigation, indicating the beneficial effects of TiO_2_ NPs (Feizi et al., 2020; Gohari et al., 2020; Dinler et al., 2024).

The conventional methods used for assessing changes such as in seed germination percentages, root elongation and nutrient uptake typically involve invasive sampling methods and extended observation period, generally ranging from 72 hours (h) to a few days after germination. However, these limitations highlight the need for a rapid, non-invasive technique capable of detecting early biological responses to NP exposure.

Optical Coherence Tomography (OCT) is a high-resolution imaging technique widely used in medical fields, but it also holds potential in plant sciences for non-invasive disease detection and seed germination assessment. Cadondon et al. utilized the TD-OCT for monitoring the bamboo plant leaf health (Cadondon et al., 2024). Similarly, another study by Shiina et al. measured the cell structure of the plant surface, its change due to the water content, and the growth monitoring of the plant tissue. This method reveals structural changes in tissue, such as alterations in cell organization and surface morphology (Shiina et al., 2005). Despite these advances, conventional OCT primarily captures structural images and does not fully exploit dynamic speckle patterns that indicate internal seed structural dynamics. Biospeckle optical coherence tomography (bOCT), developed by our group, addresses this limitation by leveraging speckle dynamics to monitor plant responses in real time with high sensitivity. Previous work applied bOCT to study the effects of acid mine drainage on rice seeds and heavy metal stress on plant tissues (Srimal et al., 2015; De Silva et al., 2021; Li et al., 2022b).

Alongside concerns about nanomaterials such as TiO_2_ NPs, plastic-derived micropollutants, such as polyethylene microplastics (PEMPs), are increasingly recognized as emerging environmental contaminants. Their widespread presence poses potential toxicity risks to plants. PEMPs are also prevalent in agricultural settings due to the widespread use of plastic materials like mulch films, irrigation pipes, and plastic-containing fertilizers and compost, which lead to microplastic contamination affecting soil functions and microbial communities (Sheng et al., 2024; Tiwari et al., 2024). Our previous work using bOCT revealed that PEMPs negatively affect lentil seed internal activity at 10, 50, and 100 mg/L concentrations, supporting other reports of their phytotoxicity (De Silva et al., 2022). However, less is known about how PEMPs interact with other common environmental NPs, such as TiO_2_ NPs, which are also prevalent in agricultural settings. Understanding these interactions is essential for evaluating combined pollutant risks in agricultural ecosystems.

Despite growing concern about TiO_2_ NPs and PEMPs, no study has systematically examined their combined effects on seeds using a rapid, non-invasive method. This study addresses this gap by validating bOCT for early detection of NP-induced responses in lentil seeds. We have chosen lentil seeds (*Lens culinaris*), a nutritionally rich crop crucial for Asian food security and economies. Lentils are ideal for studying NPs-seed interactions due to their rapid germination, distinct seed coat, and adaptability to various conditions. We compared bOCT results with conventional measurements, including seedling growth and biomass. Physiological responses such as SOD, CAT, and H_2_O_2_ activity were assessed after seven days of exposure.

## 2 Materials and methods

### 2.1 Characteristics of nanoparticles

In this experiment, lentil seeds were exposed to TiO_2_ NPs and microparticles (MPs) at concentrations of 0, 25, 100, and 200 mg/L (Laware and Raskar, 2014; Hu et al., 2020). TiO_2_ NPs were sourced from Merck Sigma–Aldrich (Tokyo, Japan). Three size variants were used: nanopowder with a primary particle size of ~21 nm (≥99.5% trace metals basis), rutile nanopowder<100 nm (≥99.5% trace metals basis), and rutile powder<5 µm (≥99.9% trace metals basis). All materials were research-grade and supplied in dry powder form (CAS numbers: 13463−67−7 and 1317−80−2). The PEMPs, of size 744–4990 nm, were purchased from Cospheric LLC (Santa Barbara, California 93160, USA). To promote uniform dispersion and address the hydrophobic nature of the particles, a 0.05% Tween-80 solution was employed as a non-ionic, biocompatible surfactant. The dispersion protocol involved initial stirring with a magnetic stirrer for 30 minutes, followed by sonication at 28 °C for 20 minutes. This process was repeated until a homogeneous dispersion was achieved, ensuring the absence of particle clusters in the solutions.

### 2.2 Plant material and experiment procedure

**Figure 1:**
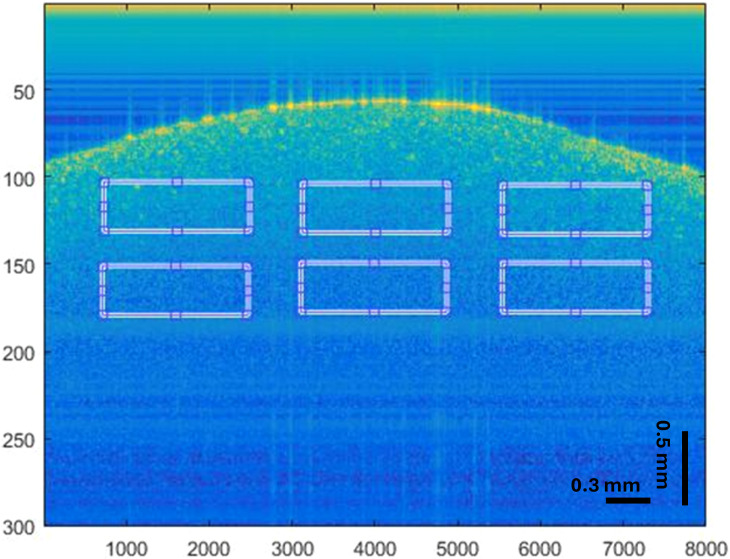
Illustrative bOCT image of a lentil seed showing the selection of six regions of interest (ROIs) used for biospeckle analysis.

Lentil seeds (*Lens culinaris*) were obtained from Greenfield Project Co. Ltd., Tokyo, Japan, a certified supplier of organic seeds, and stored in a cool and dry environment until use. Seeds weighing approximately 30 mg each were carefully selected and disinfected to ensure optimal germination and to remove surface contaminants. The disinfection process involved soaking the seeds in a 2.5% hydrogen peroxide solution for 10 minutes, followed by rinsing them three times with distilled water, to ensure growth under aseptic conditions. The seeds were then treated with TiO_2_ dispersions at concentrations of 25, 100, and 200 mg/L, and PEMPs in 1:1 and 1:2 of concentration ratios at concentrations of 25 and 100 mg/L, with distilled water used as a control. Studies have shown that at such low concentrations, Tween-80 (0.05%) does not affect the toxicity or biological impact of NPs on plants. For example, Kobayashi et al. (2019) demonstrated effective dispersion of TiO_2_ NPs in Tween-80 without adverse effects on plant toxicity assay (Kobayashi et al., 2019). Therefore, the choice of distilled water as a control was intended to avoid additional variables while ensuring proper comparison. For the bOCT experiments, six seeds were placed in each Petri dish containing 6 mL of the respective TiO_2_ dispersion and incubated in a growth chamber (MLR-351H, SANYO Electric Co., Ltd., Osaka, Japan) equipped with fluorescent lamps. In our study, each treatment condition included six seeds, and for each seed, six regions of interest (ROIs) were analyzed using bOCT to be described in sections 2.3 (**Figure 1**). Measurements were repeated three times for consistency. While these repeated measurements improve reliability, they are considered technical replicates rather than independent biological replicates. The growth chamber was maintained under controlled conditions with day/night temperatures of 25 °C/20 °C, light intensity ranging from 260-370 μmol m^−2^ s^−1^ during the day and 0 μmol m^−2^ s^−1^ at night, relative humidity between 50-65%, and a 12-hour light/dark cycle. To assess the initial response to TiO_2_ exposure, bOCT contrast images were captured at intervals of 0, 5, 10, and 20 h after treatment with NPs or MPs.

### 2.3 Biospeckle optical coherence tomography

#### 2.3.1 bOCT experimental system

**Figure 2:**
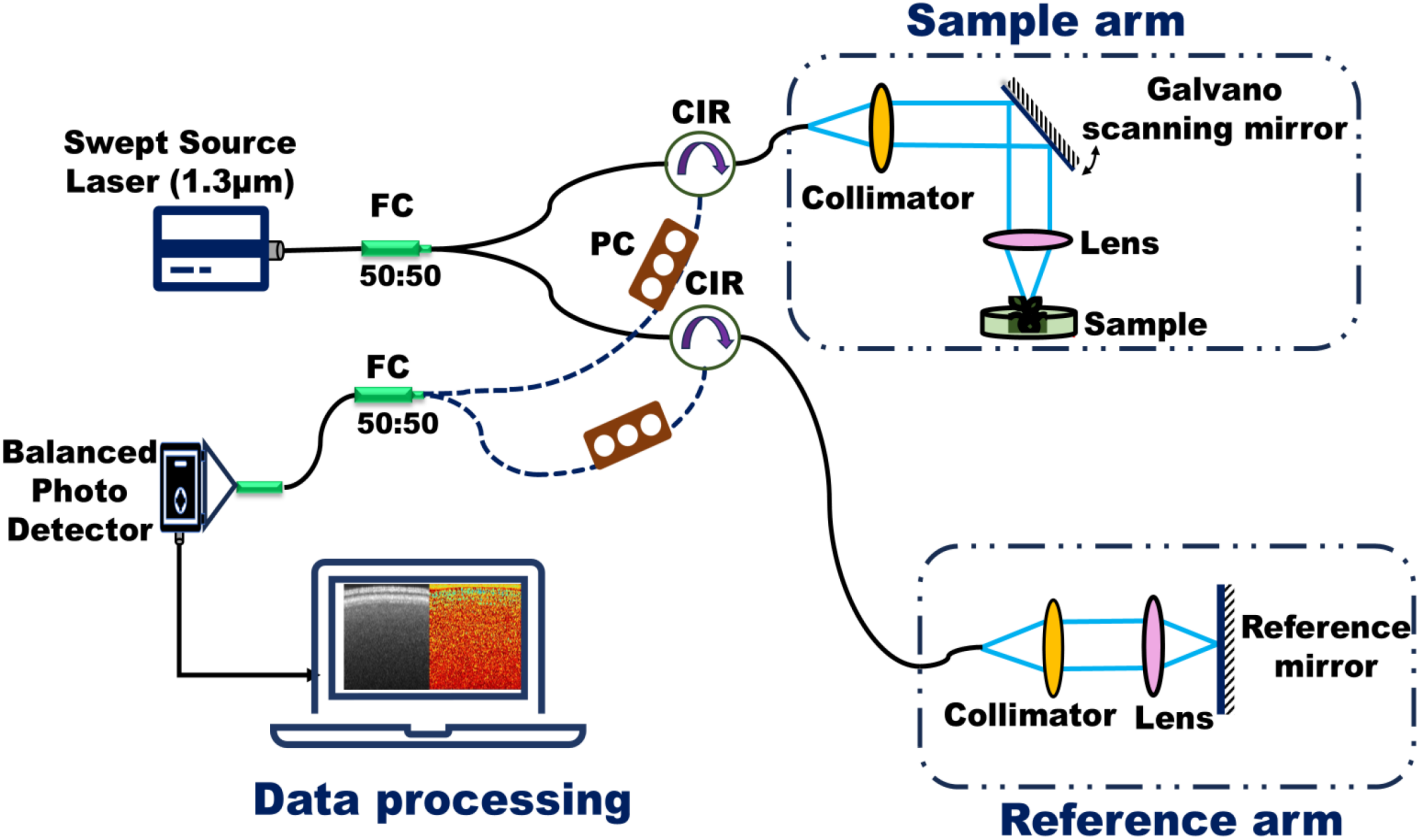
Swept-Source Optical Coherence Tomography (SS-OCT) system same as used in our earlier study (Tyagi et al., 2025) (FC: Fiber Coupler, CIR: Circulator, PC: Polarization Controller). The system employs a swept-source laser centered at 1310 nm wavelength with a 100 kHz sweep rate. Light from the source is divided by FC into the sample and reference arms. CIR directs the backscattered light from sample and reference arm to the detection unit.

The bOCT experimental setup, shown schematically in **Figure 2**, is based on a swept-source optical coherence tomography (SS-OCT) system utilizing a Mach-Zehnder interferometer and is the same employed in our earlier study of the visualization of ZnO NPs and MPs impact on lentils (Tyagi et al., 2025). For clarity, we provide the details here. It employs a swept-source laser (HSL-2100-ST, Santec, Aichi, Japan) with a central wavelength of 1.3 µm, a bandwidth of 125 nm, an average power of 23.4 mW, and a sweep frequency of 20 kHz. The system has an axial resolution of 6 µm and a lateral resolution of 39 µm. Light from the source is divided into reference and sample arms using a 50/50 optical coupler (TW1300R5A1, Thorlabs, Newton, NJ, USA). Reflected light from both arms is recombined through optical circulators (CIR-1310-50-APC, Thorlabs) and detected by a balanced photo detector (PDB470C, Thorlabs). A stepper motor stage (KXL06300-C2-F6A, Suruga Seiki, Shizuoka, Japan) adjusts the reference mirror to modify the optical path length for interference signal acquisition. In the sample arm, light is collimated and focused onto the specimen using an OCT probe unit, while galvano scanning mirrors enable lateral scanning in x-y directions to generate structural images (Santec, Aichi, Japan). Data acquisition is performed using custom LabVIEW software (LabVIEW v2012, National Instruments, Austin, USA), followed by image processing in MATLAB (MathWorks vR2016b, Natick, USA). During imaging, only the x-axis is scanned for 2D B-scans, capturing 100 frames at 12.5 frames per second over 8 seconds, with each frame containing 3048 × 1000 pixels. The spectral interference signal is converted from wavelength to k-space and Fourier-transformed to obtain depth profiles. The system uses near-infrared light at 1.3 µm with an incident power of approximately 100 µW and a radiant dose of 1.39 mJ/cm² over an 8-second exposure period, which is significantly below levels known to cause damage to plant tissues. Studies such as those by Hasan et al., which used a He-Ne laser (632.8 nm) and irradiation power of 4 mW/cm² (420 mJ/cm²) up to 105 seconds on maize seeds without adverse effects, support the assumption that this exposure is safe for seeds (Hasan et al., 2020).

#### 2.3.2 Biospeckle and contrast

When laser light illuminates a plant, it creates a speckle pattern known as biospeckle due to the scattering of light by the plant’s optically rough structures. This pattern arises from the random interference of scattered light (Goodman, 1975; Goodman, 2020). For stationary objects, the intensity of this speckle pattern remains unchanged over time. In contrast, dynamic objects, where scattering centers, such as organelles within the seed, are constantly in motion, exhibit changes in the observed intensity. The dynamic processes within the seed could be cytoplasmic flow, cell division, water transport, and cell expansion. These fluctuations in intensity are referred to as biospeckle and can be used to assess internal seed structural dynamics. In bOCT structural images, these variations are represented as intensity changes at each pixel over time, reflecting internal cellular activities. The degree of fluctuation is quantified by biospeckle contrast (*γ*), calculated as the ratio of the standard deviation of intensity to its mean value at each pixel along the temporal axis throughout the scan. This contrast is determined using the following equation (Equation 1).

(1) $$\gamma \;\left(x,z\right)=\frac{1}{<I_{OCT}\left(x,z\right)>}{\left[\frac{1}{N}\;{\sum_{j=1}^{N}\left\{I_{OCT}\left(x,z,\;t_{j}\right)-\;<I_{OCT}\left(x,z\right)>\right\}}^{2}\right]}^{\frac{1}{2}},$$

Where,


$$<I_{OCT}\left(x,z\right)>\;=\;\frac{1}{N}\sum_{j=1}^{N}I_{OCT}\left(x,z,\;t_{j}\right)\;,$$

In the given equation, *x* stands for the lateral coordinate, *z* for the depth coordinate, *j* is the scan number, *t_j_*is the time corresponding to the *j^th^* scan, and *N* indicates the total number of scans. A higher biospeckle contrast value reflects greater temporal intensity fluctuations, signifying increased internal activity within the seed being analyzed. On the other hand, a lower biospeckle contrast corresponds to smaller temporal fluctuations, indicating reduced internal activity. Therefore, biospeckle contrast can be effectively used as a parameter to evaluate how seeds respond to external environmental conditions.

### 2.4 Conventional plant growth and physiological measurements

#### 2.4.1 Growth indicators

Lentil seeds were considered germinated when their radicle extended ~2 mm (typically within 24 h) (Bewley et al., 2013). The software ImageJ (v1.54d, NIH, USA) was used to measure the root and shoot lengths of seedlings (10 seeds, n=3) grown from the treatment of TiO_2_ NPs (21 nm) and MPs (<5 µm) at 0 (control), 25, and 100 mg/L concentrations were measured. For conventional measurement 21 nm TiO_2_ and<5 µm MPs were selected because they represent the most distinct nano-to-micro size range and showed pronounced effects in preliminary screening making them relevant for detailed physiological analysis. Germination percentage was calculated to assess seed performance using given equation (Equation 2),

(2) $$Germination\;Percentage\;\left(\%\right)=\;\frac{\text{Germinated seeds}}{\text{total seeds}}\times 100$$

For biomass analysis, seedlings were washed, separated into roots and shoots, and weighed fresh using an analytical balance (AUX 320, UniBloc, Shimadzu Corporation, Japan). Dry weights were obtained after oven-drying (SOFW-450S, AS ONE, Japan) at 105 °C for 2 h, then at 80 °C until constant weight (72 h). These measurements provided insights into plant water content and growth responses under various TiO_2_ treatments, allowing for a comparison of bOCT findings at an early stage before germination.

#### 2.4.2 Biochemical indicators

To compare bOCT results with the conventional measurements, antioxidative enzyme activity in lentil seedlings (10 seeds, n=3) exposed to TiO_2_ NPs (21 nm) and MPs (<5 µm) was assessed at 25 °C under controlled conditions after 7 days of germination. Seedlings were exposed to concentrations of 0 (control), 25, and 100 mg/L. After rinsing, samples were homogenized in phosphate buffer (0.01 mol/L, pH 7.4) using a pre-chilled mortar and pestle in an ice bath (Chance and Maehly, 1955; Giannopolitis and Ries, 1977). The homogenate was centrifuged at 1000 g (3000 RPM) at 4 °C for 15 min, and the supernatant was used for the following analyses. A spectrophotometer (UV-Vis, V-730, JASCO Corporation, Hachioji, Tokyo, Japan), featuring a wide wavelength range of 190–1100 nm and a fixed spectral bandwidth of 1 nm, was used to measure absorbance for chromophore reactions and to monitor substrate degradation progress through UV absorbance decrease. H_2_O_2_ content was expressed as µmol/g fresh weight (FW). SOD activity was expressed as % inhibition of the WST reaction, following the kit protocol. CAT activity was expressed as U/g FW/min, where one unit represents the amount of enzyme decomposing 1 µmol of H_2_O_2_ per minute, following Aebi’s (1984) procedure (Aebi, 1984). The decline in absorbance corresponds to the decomposition of H_2_O_2_ by CAT.

### 2.5 Statistical analysis

For each treatment condition in bOCT, six seeds were sampled (n = 6 for each experimental condition) using three TiO_2_ particle sizes at concentrations of 0, 25, 100, and 200 mg/L. 100 OCT scans per seed were collected at 0, 5, 10, and 20 hours of exposure. The 20 h observation was chosen to capture early physiological responses before visible germination changes, aligning with the study’s aim of rapid detection. Initial OCT image data were obtained using custom LabVIEW software (ver.2012, National Instruments, USA). bOCT data analysis was performed using MATLAB (R2016b). Graphs and histograms were created using Origin 9.5. Statistical analysis was conducted using EXCEL (Microsoft 365, ver.2412, USA). Results are presented as mean ± standard deviation, with significant differences determined by Student’s t-test (p< 0.05).

## 3 Results

### 3.1 bOCT seed image comparison with conventional OCT images

**Figure 3:**
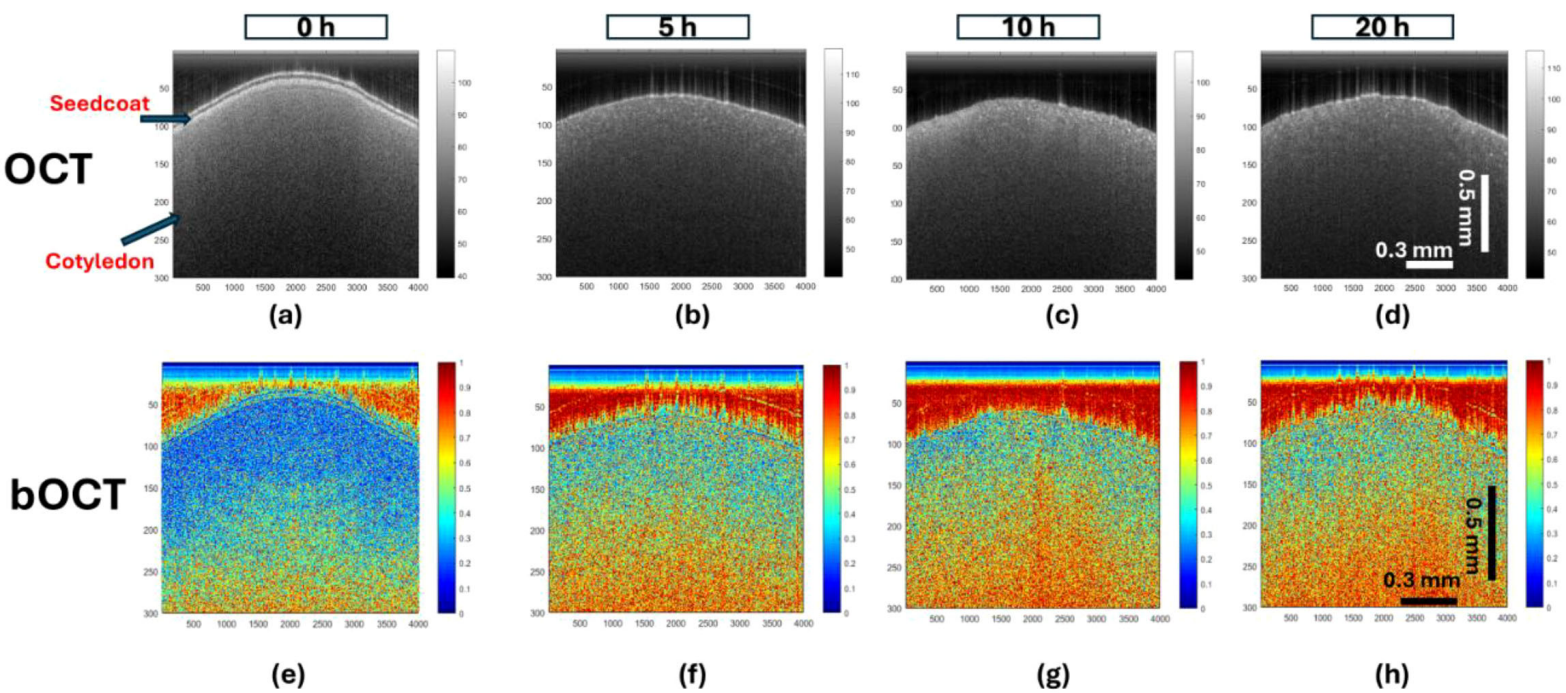
Comparison of the conventional OCT images **(a-d)** with bOCT contrast images **(e-h)** of lentil seeds exposed to TiO_2_ NPs (21 nm) at 25 mg/L. Structural OCT displays anatomical layers (bright regions: seed coat/epidermis). Axes: X-axis, Lateral position, Time (h); Y-axis = Depth. bOCT color scale: blue (low) to red (high) speckle contrast.

Lentil seeds were exposed to various concentrations of TiO_2_ NPs, followed by OCT scanning analysis. The OCT scans were acquired at 0, 5, 10, and 20 h of exposure, and bOCT images were generated to investigate internal structural changes during germination. **Figure 3** shows the temporal evolution of seed structure and activity. **Figures 3a-d** display structural OCT images with a logarithmic intensity scale, while **Figures 3e-h** show corresponding bOCT contrast images of seeds exposed to TiO_2_ NPs (21 nm, 25 mg/L) at 0, 5, 10, and 20 h. In the structural OCT images (**Figures 3a-d**), bright regions indicate high reflectivity (e.g., seed coat and epidermis), while dark areas denote weaker reflectivity. At 0 h (dry seeds), the seed coat is clearly visible; after imbibition in water or TiO_2_ NP dispersion, the coat becomes less distinguishable or detaches as the seed absorbs water, a normal physiological process. The laminar internal structure of the seed remains consistent across exposure times, with minor granular speckles attributed to subcellular structures such as mitochondria or cytoplasmic flow. The bOCT contrast images (**Figures 3e-h**), calculated using Eq. (1), reveal dynamic internal activity through color-coded biospeckle contrast. Red indicates high activity, while blue signifies low activity. For TiO_2_ NPs (21 nm, 25 mg/L), biospeckle contrast increased significantly over time (0 to 20 h), with dominant red regions at 20 h (**Figure 3h**), reflecting enhanced internal activity.

While the structural OCT images (**Figures 3a-d**) showed no visible changes, the bOCT images demonstrated increases in activity. This highlights the sensitivity of bOCT in detecting early biochemical dynamics that precede visible structural changes during seed germination under TiO_2_ NPs exposure.

**Figure 4:**
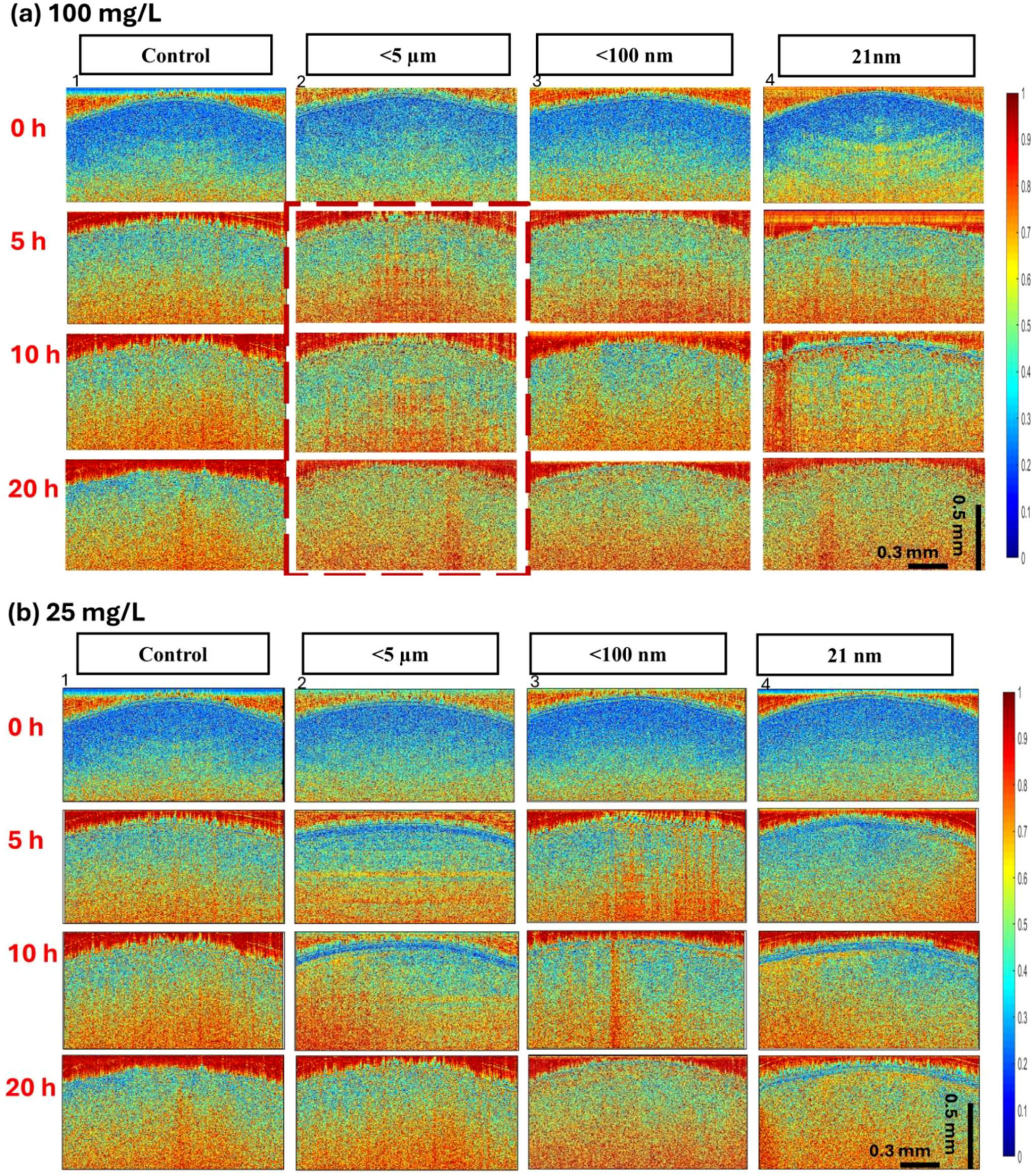
bOCT contrast image comparison of TiO_2_ NPs exposed seeds at each observation time at 0, 5, 10, and 20 h for **(a)** 100 mg/L and **(b)** 25 mg/L. Key trends: increased red color density indicates higher internal activity compared to control or earlier time points.

**Figures 4a, b** illustrate the effects of TiO_2_ particle size and concentration on lentil seed internal activity using bOCT contrast images. The images are organized in a matrix format, with rows representing exposure times (0, 5, 10, and 20 h) and columns showing different particle sizes (<5 µm,<100 nm, and 21 nm), alongside a control group treated with distilled water. **Figure 3a** displays the 100 mg/L concentration results, while **Figure 3b** shows the 25 mg/L concentration results. This arrangement allows for direct comparison of how internal seed activity changes over time across various particle sizes and concentrations.

At the higher concentration (**Figure 4a**), <5 μm particles exhibit the most pronounced red density increase, particularly at 10 and 20 h.<100 nm and 21 nm particles also show significant contrast enhancement at 100 mg/L. At the lower concentration (**Figure 4b**), the trend persists but with reduced intensity. Here,<5 μm particles maintain consistent contrast increases across all time points, while<100 nm and 21 nm particles show gradual rises, becoming more pronounced at 10 and 20 h. Interestingly, at 20 h, the 21 nm particles show a slight decrease in red color density compared to the control, while still maintaining higher activity than at earlier time points.

These results suggest TiO_2_ particles promote internal activity within seeds regardless of size or concentration, as evidenced by the increased biospeckle contrast in bOCT images. The conventional OCT structural images fail to reveal such dynamic changes, highlighting the superior sensitivity of bOCT for visualizing internal activity in seeds.

### 3.2 bOCT contrast measurements

A detailed quantitative analysis of bOCT images was conducted to investigate changes in the internal activity of seeds. This process involved computing normalized contrast and average local contrast values. Regions of interest (ROIs) were identified within both the shallow and deeper areas of the seed to calculate average local contrast. Six ROIs were selected from each bOCT image, and a grand average of the average local contrast was derived from these six ROIs (**Figure 1**). To account for individual seed variability, the average local contrast for each treatment at different time points was normalized against the values obtained at 0 h (pre-imbibition). Subsequently, the average normalized contrast for each treatment was calculated by averaging these normalized values across a sample of six seeds. This two-step approach effectively minimized seed-to-seed variability, enabling a more reliable analysis of variations in internal seed activity.

**Figure 5:**
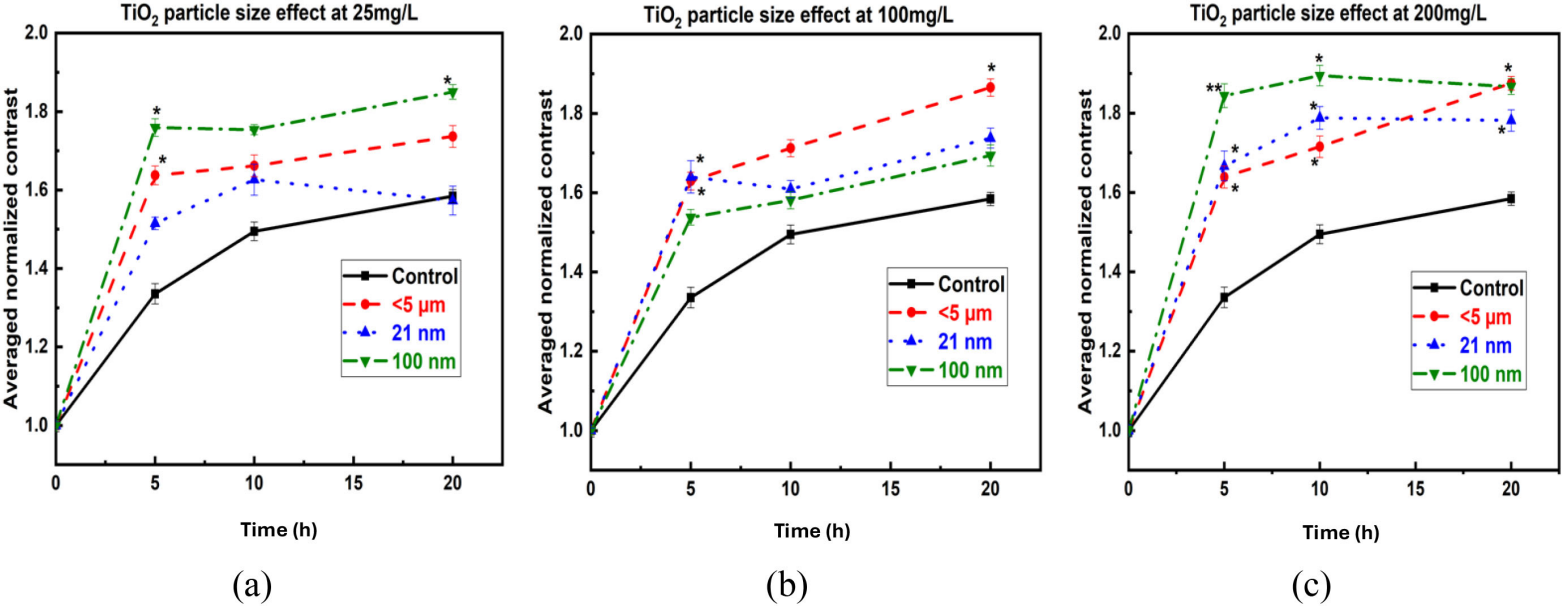
Averaged normalized biospeckle contrast of lentil seeds exposed to TiO_2_ NPs at concentrations of **(a)** 25 mg/L, **(b)** 100 mg/L, and **(c)** 200 mg/L. * and ** denote statistical significance compared to control (*p< 0.05, **p< 0.01). Legend colors/symbols indicate particle sizes: black squares = control, red circles =<5 µm, blue triangles = 21 nm, green inverted triangles = 100 nm. Error bars represent SD. Axes: X-axis = Time h; Y-axis = averaged normalized contrast value.

The calculation of averaged normalized contrast revealed distinct size-dependent effects at both lower and higher concentrations. **Figures 5a–c** show the biospeckle contrast results, where increases in contrast were already detectable after 5 h of exposure (p<0.05). In **Figure 5a** (25 mg/L), TiO_2_ particles<5 µm and<100 nm showed a prominent increase in internal seed activity, with average normalized contrast increases of 22.6% and 31.7%, respectively, at 5 h (p<0.05), while the smallest TiO_2_ particles (21 nm) did not exhibit detectable changes at this lower concentration.

**Figure 5b** (100 mg/L) demonstrates that<5 µm and 21 nm particles showed an increase in normalized contrast just at 5 h of exposure (22.0% and 22.8%, respectively), while<100 nm particles exhibited a lesser effect (15.1%). In **Figure 5c** (200 mg/L), the effect was highest for<100 nm particles (38.1% at 5 h, p<0.01), followed by 21 nm particles (24.8%, p<0.05) and<5 µm particles (22.7%, p<0.05) up to 10 h of exposure.

**Figure 6:**
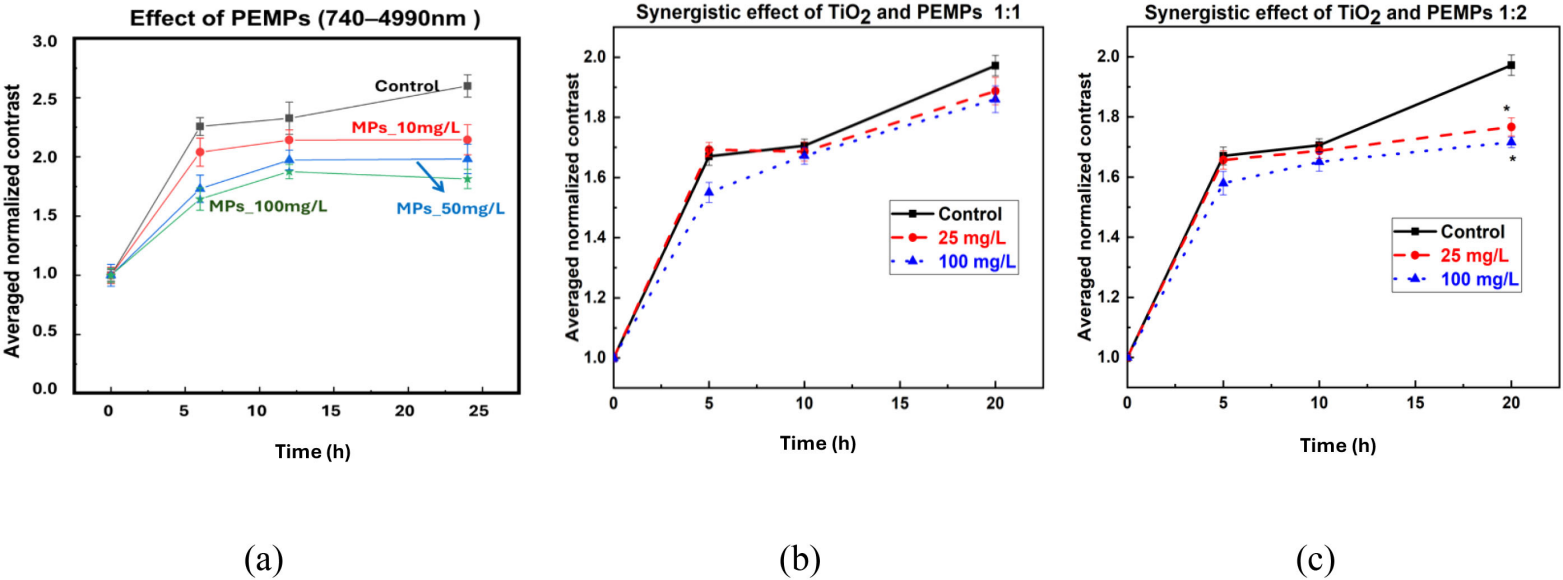
Averaged normalized biospeckle contrast of lentil seeds exposed to **(a)** microplastics (PEMPs) individually (De Silva et al., 2022), and the synergy of TiO_2_ NPs and PEMPs at concentration ratios of **(b)** 1:1 and **(c)** 1:2. * denotes statistical significance compared to control (*p< 0.05). Legend colors/symbols indicate concentration: black squares = control, red circles = 25 mg/L, blue triangles = 100 mg/L. Panel **(a)** is adapted from a previous study (De Silva et al., 2022); panels **(b, c)** show absolute values matching the scale in **Figure 4**. Axes: X-axis = Time (h); Y-axis = averaged normalized contrast value.

In addition to examining the effects of TiO_2_ NPs alone, we also investigated their synergistic interaction with PEMPs, as illustrated in **Figures 6a, b**. Our previous study by De Silva et al. found that PEMPs at concentrations of 10, 50, and 100 mg/L had a negative impact on the internal activity of lentil seeds, as shown in **Figure 6a** (De Silva et al., 2022). In **Figure 6b**, we present the combined effect of TiO_2_ NPs and PEMPs at a 1:1 ratio. Here, the negative impact of PEMPs is mitigated, as the averaged normalized contrast shows no difference compared to the control, indicating a protective or alleviating effect from TiO_2_ NPs. Conversely, when the concentration of PEMPs is doubled relative to TiO_2_ NPs (**Figure 6c**), there is a decrease in the averaged normalized contrast compared to the control (p<0.05), reflecting a stronger negative effect from PEMPs.

The proposed bOCT results suggest that TiO_2_ NPs, at both lower and higher concentrations, do not adversely affect the internal activity of lentil seeds. Moreover, TiO_2_ NPs exhibit a positive effect, as they help counteract the detrimental impact of PEMPs when present in equal concentrations.

### 3.3 Conventional measurements

Following exposure to TiO_2_ NPs and MPs, conventional morphological and physiological assessments, including germination rate, shoot length, root length, fresh and dry weight, and enzyme activity, were performed after 7 days of exposure to complement the bOCT findings. For comparative analysis, we focused on two particle sizes (21 nm and<5 μm) and two concentrations (25 and 100 mg/L), selected from the broader range of sizes and concentrations tested in the bOCT experiment. The concentrations 25 and 100 mg/L were chosen to create a pronounced contrast between lower and higher exposure levels. The particle sizes<21 nm and<5 µm were selected to represent the smallest NPs and a distinctly larger MPs fraction, thereby maximizing the size gap for assessing size-dependent effects. For reasons of experimental feasibility, not all concentration–size combinations identified as interesting by bOCT (e.g., 200 mg/L and 100 nm) could be included in the conventional assays, and only a subset of conditions was monitored physiologically. Future experiments will be designed to align measurement timing and to apply identical concentration and NPs size conditions across bOCT and conventional methods, and to use the same developmental stage, in order to enable a more direct and systematic comparison of the different measurement approaches.

#### 3.3.1 Growth indicators

**Figure 7:**
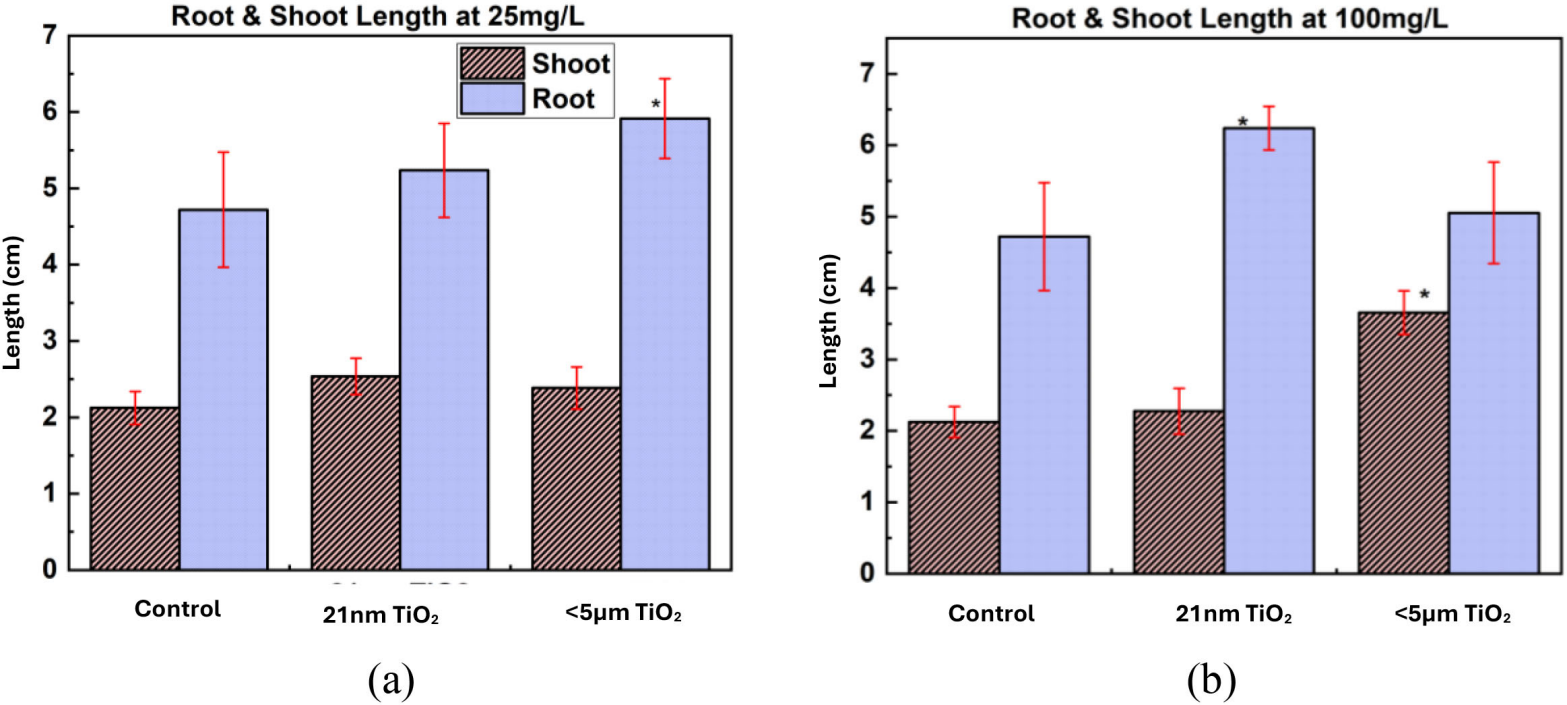
Root and shoot length exposed to TiO_2_ NPs at concentrations of **(a)** 25 mg/L, **(b)** 100 mg/L after 7 days. Statistical significance was determined by t-test; *p< 0.05, vs. control. Error bars: SD, Axis: X=Particle size, Y=Length (cm).

Root and shoot length measurements after 7 days of exposure are shown in **Figures 7a, b** for TiO_2_ NPs at 25 and 100 mg/L concentrations, respectively. At 25 mg/L (**Figure 7a**), seeds exposed to<5 μm TiO_2_ particles exhibited a significant increase in root length compared to the control (t-test, p< 0.05). At 100 mg/L (**Figure 7b**), seeds treated with 21 nm TiO_2_ particles showed a significant increase in root length. Additionally, shoot length significantly increased following exposure to 100 mg/L<5 μm TiO_2_ particles. These findings are consistent with bOCT results, which showed increased biospeckle contrast correlating with enhanced seedling growth.

Biomass measurements (fresh and dry weight of roots and shoots) did not show any statistically significant differences across TiO_2_ NPs treatments at either concentration (see **Appendix A1**, **Supplementary Figure S1** for details).

#### 3.3.2 Germination and antioxidative response

**Figure 8:**
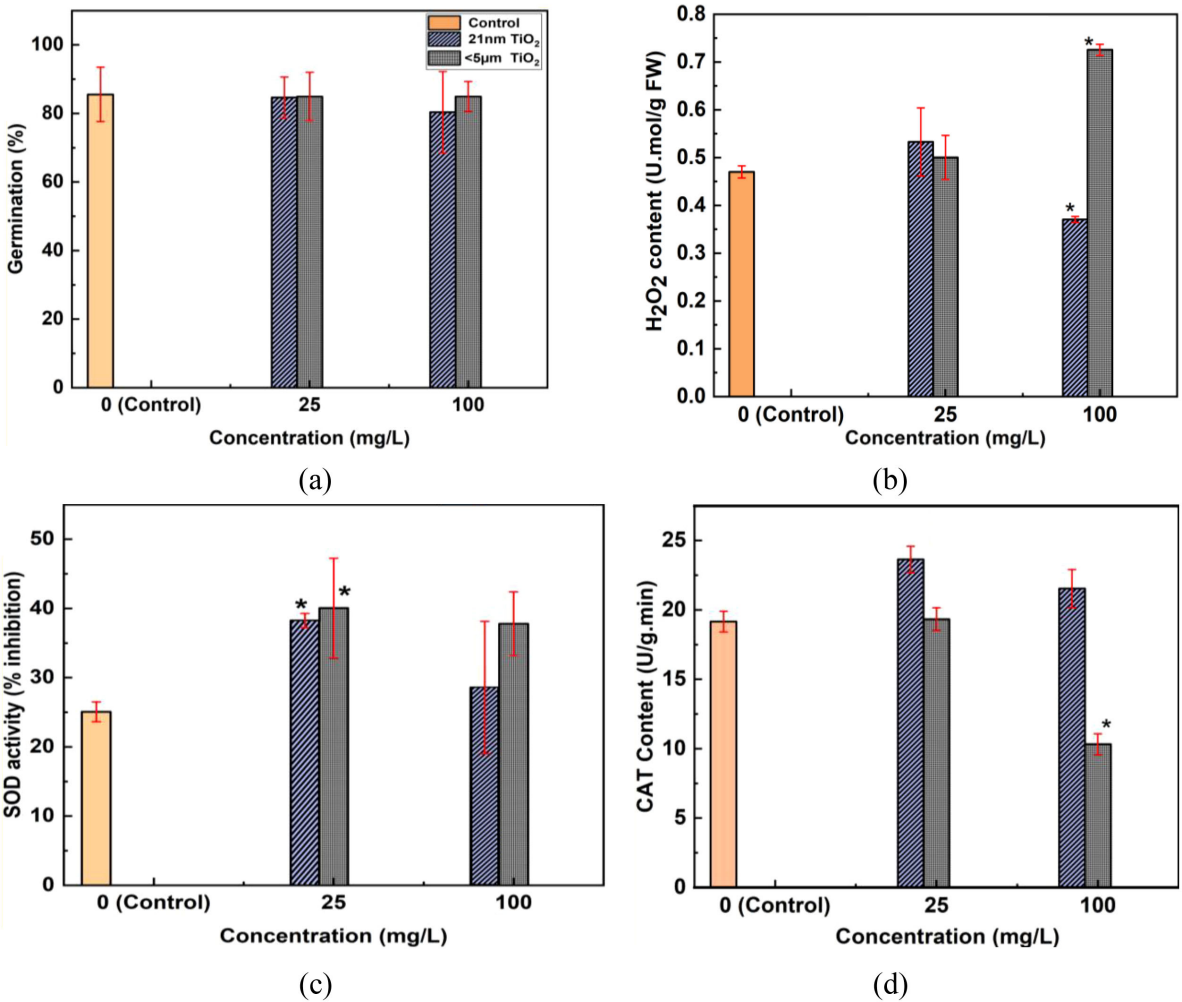
**(a)** Germination percentage (%), **(b)** hydrogen peroxide (H_2_O_2_) content (U.mol/g FW), **(c)** superoxide dismutase (SOD) activity (% inhibition), and **(d)** catalase (CAT) content (U/g·min) of lentil seedlings exposed to TiO_2_ NPs at 25 and 100 mg/L after 7 days. Axes: X-axis TiO_2_ NP concentration (mg/L); Y-axis respective parameter values as indicated. Statistical significance was determined by t-test (*p< 0.05 vs. control).

Germination percentage (%) of lentil seeds after 7 days of exposure is presented in **Figure 8a**. No significant changes in germination percentage were observed for either size of TiO_2_ NPs exposure compared to the control after 7 days.

**Figure 8b** shows the H_2_O_2_ content measurement results. A significant increase (t-test, p< 0.05) in H_2_O_2_ content was observed due to<5 μm TiO_2_ exposure, while a significant decrease was noted for 21 nm TiO_2_ NPs exposure at 100 mg/L concentration. However, at 25 mg/L concentration, no significant change in H_2_O_2_ content was observed.

Similarly, SOD activity (% inhibition) results are shown in **Figure 8c**. An increase (t-test, p< 0.05) in SOD activity was observed even at lower concentrations (25 mg/L) for both particle sizes (21 nm and<5 μm). This indicates a positive response of the plant defence mechanism, which aids in H_2_O_2_ regulation.

**Figure 8d** presents data related to CAT content, with the horizontal axis corresponding to exposure concentration and the vertical axis showing CAT content. There is no significant change (t-test, p< 0.05) in CAT content observed at 25 mg/L, while at 100 mg/L, CAT content decreased due to<5 μm TiO_2_ particle exposure. These biochemical assay results complement the bOCT findings, indicating no negative impact of TiO_2_ NPs exposure on lentil seeds.

## 4 Discussion

This study aimed to investigate the differential effects of TiO_2_ NPs of varying sizes and their synergistic interactions with PEMPs on lentil seed germination and early seedling development using bOCT. Prior work on TiO_2_ largely relied on multi-day, post-germination physiological assays, leaving a gap in rapid, non-invasive methods capable of detecting early NP-induced changes in seeds. By leveraging bOCT for real-time internal activity assessment, our study addresses this methodological gap and provides a framework for linking early internal seed dynamics to conventional outcomes. This technique provides an indirect measure of physiological changes through speckle dynamics but does not allow direct visualization of NPs penetration or accumulation. Although TiO_2_ NPs scatter light strongly in the visible range (400–700 nm), their scattering effect at 1.3 µm (the wavelength used in our bOCT system) is minimal, so biospeckle signals primarily reflect seed structural dynamics rather than optical artifacts (Perera et al., 2012; Tsai et al., 2016).

Since speckles originate from the interference of scattered light, it is important to consider the nature of the scatterers within the seed. Scattering arises from both biological organelles and NPs, but the dominant contribution depends on their relative size to the wavelength. Organelles, being comparable to or larger than the wavelength, exhibit Mie scattering, whereas smaller TiO_2_ NPs mainly produce weak Rayleigh scattering (Bohren and Huffman, 1983). In the OCT system, the backscattered components within the coherence volume primarily originate from organelles undergoing physiological motion or refractive index fluctuations. Therefore, dynamic speckle changes are more consistent with biological activity than with the static presence of NPs.

Future studies incorporating imaging and elemental mapping methods (e.g., SEM EDX, TEM) are recommended to complement bOCT findings.

A summary of the effects of TiO_2_ NPs and MPs at 25 and 100 mg/L on lentil by bOCT measurements (20 h) and the conventional physiological and biochemical parameters (7 days) is presented in **Table 1**. The table provides an overview of the observed changes relative to the control across all measured endpoints.

**Table 1:** Summary of the effects of TiO_2_ NPs and MPs at 25 and 100 mg/L on bOCT (measured after 20 h) and the conventional physiological and biochemical parameters (measured after 7 days) in lentil seeds.

Particle type (TiO_2_)	Conc. (mg/L)	bOCT contrast (20 h)	Growth parameters (7 d)			Antioxidant enzymes (7 d)		
			Root length	Shoot length	Germination %	CAT	SOD	H_2_O_2_
Nano (21 nm)	25	Increase	Increase	Increase	No change	Increase	Increase*	No change
	100	Increase	Increase*	No change	No change	No change	No change	Decrease*
Micro (<5 µm)	25	Increase	Increase*	No change	No change	No change	Increase*	No change
	100	Increase*	Increase	Increase*	Increase	Decrease*	Increase	Increase

Asterisk (*) indicates a statistically significant difference compared to the control (t-test, *p< 0.05).

Effects are shown relative to the control.

Unlike the conventional OCT, which primarily focuses on structural images, bOCT offers the unique capability to monitor dynamic changes within seeds during germination in real-time and *in vivo*. This allows for a more comprehensive understanding of how NPs influence seed development over significantly shorter periods than traditional methods. Our study observed significant responses within just 20 h, with cumulative TiO_2_ exposure doses (calculated as concentration × time; mg·L^−1^·h) ranging from 500 to 4000 mg·L^−1^·h. This is substantially faster than the typical 7-day observation period used in many studies, which corresponds to broad cumulative dosage ranges of 168 to 33,600 mg·L^−1^·h, depending on exposure concentrations, and demonstrates bOCT’s sensitivity in detecting early biological responses to NPs, highlighting its potential as a valuable tool for studying plant-NP interactions.

The bOCT results revealed distinct size and concentration-dependent effects of TiO_2_ particles on lentil seed internal activity. Interestingly, no adverse effects were observed even at the highest tested concentration (200 mg/L), indicating the biocompatibility of TiO_2_ NPs across all sizes. At 25 mg/L, particles<100 nm induced the most significant increase in averaged normalized contrast, followed by<5 µm particles, whereas 21 nm NPs showed no significant difference compared to the control. This size-dependent effect aligns with our previous study on ZnO NPs and MPs, where 100 nm particles exhibited the most pronounced negative impact, surpassing even the smallest<50 nm particles (Tyagi et al., 2025). Similarly, in this study, we observed that<100 nm TiO_2_ particles had a more positive effect than 21 nm particles, suggesting a consistent trend in size-dependent NPs interactions with lentil seeds and aggregation tendency of ultra-small NPs (Xu et al., 2023). The discrepancy in particle effects may relate to lentil seed pore sizes (4–8 µm), which likely enable smaller particles (<100 nm) to penetrate and interact directly with internal tissues (Aouaini et al., 2015). All particle sizes significantly enhanced contrast values at higher concentrations (200 mg/L), suggesting that increased concentrations overcome potential aggregation limitations.

Root and shoot length measurements corroborated the bOCT findings, showing significant increases in growth parameters. These results align with studies reporting improved seedling growth in lentils exposed to TiO_2_ NPs, attributed to enhanced water uptake and nutrient absorption (Reddy et al., 2018; Azimi, 2020). Biomass measurements further supported the non-toxic nature of TiO_2_ particles at the tested concentrations. The slight increase in root fresh weight at 25 mg/L for 21 nm particles suggests improved water uptake efficiency.

Oxidative stress responses showed dose- and size-specific patterns that warrant careful interpretation. At lower concentrations, both particle sizes increased superoxide dismutase (SOD) activity, indicating a controlled activation of antioxidant defense. The reduction in H_2_O_2_ by 21 nm NPs at 100 mg/L suggests potential ROS scavenging or modulation by smaller particles (Yang et al., 2024). Importantly, the co-occurrence of increased SOD and H_2_O_2_ with reduced catalase (CAT) activity observed for<5 μm TiO_2_ at 100 mg/L represents a classical “paradox”: while elevations in H_2_O_2_ and reductions in CAT are canonical indicators of oxidative stress, the simultaneous enhancement in growth suggests a stress-induced morphogenic response (SIMR) rather than damaging oxidative stress. In SIMR, H_2_O_2_ functions as a signaling molecule that facilitates cell wall loosening and root elongation (Potters et al., 2007; Tian et al., 2018; Mohammadi et al., 2023). Thus, our interpretation favors “controlled, positive stress” under these exposure conditions; however, we acknowledge that such profiles can indicate oxidative stress in other contexts, underscoring the value of integrating biochemical signals with growth outcomes.

Furthermore, this study revealed that TiO_2_ NPs have the potential to alleviate the negative effects of PEMPs on lentil seeds when both substances are applied at equal concentrations (1:1). This finding is particularly significant given that our earlier study by De Silva et al. showed that PEMPs alone exert a pronounced negative impact on lentil internal activity. However, when the concentration of PEMPs was increased to twice that of TiO_2_ NPs, this mitigating effect was lost, and a significant negative impact was observed, highlighting the dose-dependent toxicity of PEMPs in plants (De Silva et al., 2022). A plausible mechanism is that TiO_2_ enhances light absorption and energy conversion, protects chloroplast integrity, improves nitrogen metabolism, and strengthens antioxidant capacity (Cherchi and Gu, 2010; Ze et al., 2011; Moshirian Farahi et al., 2023; Yang et al., 2024). Hence, buffering PEMPs-induced oxidative and metabolic disruption at 1:1. At 1:2, the PEMPs burden likely exceeds the compensatory capacity conferred by TiO_2_, revealing dose-dependent toxicity and the limits of NP-mediated mitigation.

In conclusion, this study demonstrates the potential of TiO_2_ particles as plant growth promoters in lentil seeds, with a complex interplay between particle size, concentration, and physiological responses. The consistency between bOCT results and the conventional growth parameters underscores the technique’s value in rapidly assessing NP effects on seed metabolism. Significant changes were recorded at 0, 5, 10, and 20 h, with control samples showing a consistent increase over time. This highlights bOCT’s sensitivity to biological processes within the seeds, with variations reflecting physiological responses rather than NP motion artifacts caused by NPs. Similar findings include bOCT-based monitoring of acid mine drainage (AMD) exposure effects on Kaiware daikon and soybean seeds by Li et al (De Silva et al., 2022; Li et al., 2022a).

Furthermore, in our earlier study on ZnO NPs demonstrated dose-dependent effects on internal activity and germination, reinforcing bOCT’s ability to detect NP-induced physiological changes non-invasively. These findings support bOCT’s robustness in monitoring seed activity under diverse stressors. Observed biospeckle contrast alterations during exposure directly correlate with physiological responses to TiO_2_ NPs, highlighting bOCT’s potential for early detection of NP effects before visible germination. Its real-time monitoring capability also provides unique insights into how environmental stressors influence plant development.

## 5 Conclusion

This study demonstrates the effective application of bOCT as a rapid, non-invasive tool for monitoring the effects of TiO_2_ NPs on the internal activity of lentil seeds. Significant changes in seed–NP interactions were detected within only 20 h of exposure, underscoring the technique’s sensitivity and speed compared to traditional methods requiring multi-day observations. The results revealed that smaller TiO_2_ NPs (<100 nm) at lower concentrations (25 mg/L) had the most pronounced impact on internal activity, likely due to their ability to penetrate seed pores (4–8 µm) and interact directly with internal tissues. Larger particles (<5 µm) also promoted internal activity, particularly at higher concentrations (100 mg/L), while the smallest particles (e.g., 21 nm) at low concentrations showed less bioavailability. These size-dependent effects were reflected in germination rates, seedling growth, and antioxidative enzyme activities. Synergistic effects observed during co-exposure with PEMPs at equal concentrations (1:1) included no significant difference in contrast value compared to the control, highlighting TiO_2_ NPs as stress mitigators. However, this protective effect was lost at 1:2 ratios, indicating dose-dependent toxicity of PEMPs and the limits of NP-mediated mitigation. These findings are consistent with previous research on ZnO particles, suggesting that particle size plays a crucial role in governing bioavailability and biological impact in plant–NP interactions.

Although TiO_2_ NPs enhanced seedling vigor in this study, potential environmental risks such as NP accumulation in soil and entry into the food chain warrant further investigation. Future research should focus on optimizing NP size and concentration for sustainable crop enhancement while evaluating ecological safety and scalability. Additionally, while bOCT offers unique advantages for real-time, non-invasive monitoring, its performance may be limited by light penetration in thicker seeds and variability due to tissue composition, which should be addressed in future studies.

Overall, integrating bOCT into agricultural research provides a promising approach for rapid screening of NP effects, supporting the development of sustainable, nano-enabled crop enhancement strategies while ensuring environmental responsibility.

## Data Availability

The raw data supporting the conclusions of this article will be made available by the authors, without undue reservation.

## Supplementary material

Supplementary Figure 1 **(a, b)** Fresh weight; **(c, d)** Dry weight of lentil seedlings exposed to TiO_2_ NPs at 25 and 100 mg/L, respectively.

## Appendix A1. Biomass measurements of lentil seedlings exposed to TiO_2_ NPs.

**Supplementary Figures S1a-d** show root and shoot fresh weight (FW) and dry weight (DW) of seedlings exposed to TiO_2_ NPs at 25 and 100 mg/L. The horizontal axis represents particle size, and the vertical axis shows weight (g). No statistically significant differences were observed in FW or DW among treatments (t-test, p > 0.05). At 25 mg/L (**Supplementary Figure S1a**), a slight increase in root FW was observed for 21 nm particles, while at 100 mg/L (**Supplementary Figure S1b**), lower FW of roots and shoots was noted for 21 nm particles compared to the control. However, none of these differences were statistically significant. DW measurements (**Supplementary Figures S1c, d**) also showed no significant changes.
