# Supplementary figures and images for: Synergistic effects of titanium dioxide nanoparticles and microplastics on lentil seeds by a non-invasive biospeckle optical coherence tomography
Source: Front Plant Sci. 2026 Feb 23;17:1718010. doi: 10.3389/fpls.2026.1718010 (PMC12968291; doi:10.3389/fpls.2026.1718010)

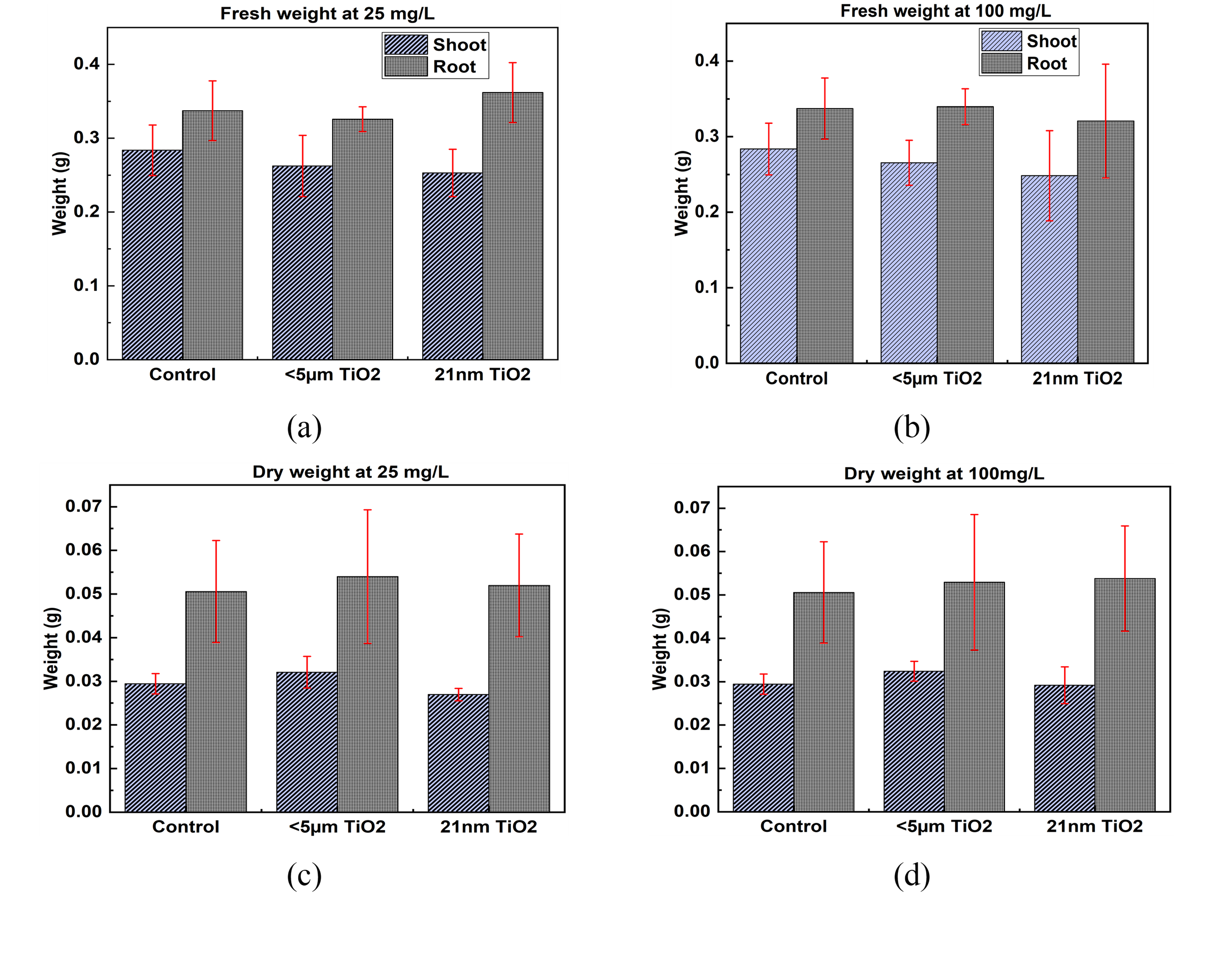

Supplement: Supplementary Figure 1 — (a, b) Fresh weight; (c, d) Dry weight of lentil seedlings exposed to TiO2 NPs at 25 and 100 mg/L, respectively. [file Image1.png]
